# A Set-Based Mixed Effect Model for Gene-Environment Interaction and Its Application to Neuroimaging Phenotypes

**DOI:** 10.3389/fnins.2017.00191

**Published:** 2017-04-06

**Authors:** Changqing Wang, Jianping Sun, Bryan Guillaume, Tian Ge, Derrek P. Hibar, Celia M. T. Greenwood, Anqi Qiu, the Alzheimer's Disease Neuroimaging Initiative

**Affiliations:** ^1^NUS Graduate School for Integrative Sciences and Engineering, National University of SingaporeSingapore, Singapore; ^2^Department of Epidemiology, Centre for Clinical Epidemiology, Lady Davis Institute for Medical Research, Jewish General Hospital, McGill UniversityMontreal, QC, Canada; ^3^Department of Biomedical Engineering, National University of SingaporeSingapore, Singapore; ^4^Athinoula A. Martinos Center for Biomedical Imaging, Harvard Medical School, Massachusetts General HospitalBoston, MA, USA; ^5^Psychiatric and Neurodevelopmental Genetics Unit, Center for Human Genetic Research, Massachusetts General HospitalBoston, MA, USA; ^6^Imaging Genetics Center, Institute for Neuroimaging and Informatics, Keck School of Medicine of the University of Southern CaliforniaLos Angeles, CA, USA; ^7^Departments of Oncology, Epidemiology, Biostatistics and Occupational Health, and Human Genetics, McGill UniversityMontreal, QC, Canada; ^8^Clinical Imaging Research Centre, National University of SingaporeSingapore, Singapore; ^9^Singapore Institute for Clinical Sciences, Agency for Science, Technology, and ResearchSingapore, Singapore

**Keywords:** gene-environment interaction, Alzheimer's disease, imaging genetics, score statistics, tensor-based morphometry

## Abstract

Imaging genetics is an emerging field for the investigation of neuro-mechanisms linked to genetic variation. Although imaging genetics has recently shown great promise in understanding biological mechanisms for brain development and psychiatric disorders, studying the link between genetic variants and neuroimaging phenotypes remains statistically challenging due to the high-dimensionality of both genetic and neuroimaging data. This becomes even more challenging when studying gene-environment interaction (G×E) on neuroimaging phenotypes. In this study, we proposed a set-based mixed effect model for gene-environment interaction (MixGE) on neuroimaging phenotypes, such as structural volumes and tensor-based morphometry (TBM). MixGE incorporates both fixed and random effects of G×E to investigate homogeneous and heterogeneous contributions of multiple genetic variants and their interaction with environmental risks to phenotypes. We discuss the construction of score statistics for the terms associated with fixed and random effects of G×E to avoid direct parameter estimation in the MixGE model, which would greatly increase computational cost. We also describe how the score statistics can be combined into a single significance value to increase statistical power. We evaluated MixGE using simulated and real Alzheimer's Disease Neuroimaging Initiative (ADNI) data, and showed statistical power superior to other burden and variance component methods. We then demonstrated the use of MixGE for exploring the voxelwise effect of G×E on TBM, made feasible by the computational efficiency of MixGE. Through this, we discovered a potential interaction effect of gene *ABCA7* and cardiovascular risk on local volume change of the right superior parietal cortex, which warrants further investigation.

## 1. Introduction

Neurodegenerative diseases such as Alzheimer's disease (AD) are highly heritable, but how specific genetic variants contribute to these diseases remains largely undetermined (Pedersen et al., 2004; Bertram et al., 2010; Ridge et al., 2013). Neuroimaging has received great attention for understanding the genetic contribution to psychiatric disorders. It provides attractive intermediate phenotypes, including brain morphology, functional activity, and brain wiring, *etc*, that are closer to the biology of genetic function than clinical phenotypes of illnesses or cognitive phenotypes (Hariri et al., 2006; Mattay et al., 2008; Bigos and Weinberger, 2010). Imaging genetics has thus become an emerging field for the investigation of neural mechanisms underlying genetic variation. Imaging genetics aims to determine how differences in single nucleotide polymorphism (SNP) lead to differences in brain anatomy and function, and hence to understand how variants in SNPs lead to diseases. Although imaging genetics has recently shown great promise in the domains of studying brain development (Viding et al., 2006; de Geus et al., 2008; Mattay et al., 2008; Rasch et al., 2010), as well as psychiatric disorders (Hariri et al., 2006; Meyer-Lindenberg and Weinberger, 2006; Domschke and Dannlowski, 2010; Durston, 2010; Scharinger et al., 2010; Tost et al., 2012), studying the link between genetic variants and neuroimaging phenotypes remains statistically challenging, especially when dealing with high dimensional neuroimaging data, such as tensor-based morphometry, whole brain functional activity, *etc*. This becomes even more challenging when studying the effect of G×E on neuroimaging phenotypes.

Genome-wide association studies (GWAS) is a simple and widely used technique to determine associations between genetic polymorphisms and neuroimaging phenotypes, such as structural volumes (Stein et al., 2012). For GWAS, each SNP is independently tested for association with a scalar measure using regression models. There are however some limitations to genome-wide association studies (GWAS). Common genetic variants only account for a small fraction of the heritability of brain phenotypes such as hippocampal and subcortical volumes (Stein et al., 2012; Hibar et al., 2015). “Missing,” or perhaps “hidden,” heritability may be accounted for by rare variants (Korte and Farlow, 2013), which are not even considered in GWAS due to the lack of statistical power (Lee et al., 2014). Population genetic theory supports the idea that rare variants may play a significant role in brain-related diseases (Manolio et al., 2009). But rare variants can only be identified using GWAS with a large sample size (Manolio et al., 2009), which is difficult to achieve in imaging genetics. In fact, even common variants do not usually pass genome-wide significance if they have very small effect sizes, after adjusting for the very large number of variants that are tested (Gibson, 2010). GWAS becomes even more difficult if voxelwise neuroimaging measures are used as phenotypes, such as voxelwise GWAS (Stein et al., 2010; Hibar et al., 2011; Ge et al., 2012; Huang et al., 2015), due to computational cost and the lack of statistical power.

Set-based association tests are an alternative class of techniques that are able to overcome the aforementioned limitations of GWAS with regards to rare variants or common variants with small effect sizes. In contrast to GWAS, this class of techniques examines whether a set of genetic variants is collectively associated with a phenotype. The variant set can be defined as variants that belong to a particular gene, pathway, or any other biologically meaningful systems. With a set-based association test, genetic effects of individual variants are accumulated within the set into a single large effect, such that the set could be significantly associated with phenotype even if the individual variants are not. Furthermore, a set-based association test reduces the number of independent statistical tests conducted, which implies less stringent correction for multiple comparisons than that for GWAS. Lee et al. (2014) reviewed five different types of set-based association tests, including burden tests, adaptive burden tests, variance component tests, combined tests, and Exponential Combination test. Burden tests, such as Cohort Allelic Sum Test (Morgenthaler and Thilly, 2007) and Weighted Sum Statistic (Madsen and Browning, 2009), collapse variants into a burden score, and are more powerful when a large proportion of the variants in a set is causal, and the effects are homogeneous (equally deleterious for example). Adaptive burden tests, such as Data-adaptive Sum Test (Han and Pan, 2010) and Variable Threshold (Price et al., 2010), use data adaptive weights, and are more powerful than burden tests but are computationally intensive. Variance component tests, such as C-alpha test (Neale et al., 2011) and sequence kernel association test (SKAT) (Wu et al., 2011), examine the variance of genetic effects, and are more powerful when the effects of individual genetic variants are heterogeneous, even if the variants have opposing directions of effects. Combined tests – such as Optimal SKAT (Lee et al., 2012), Fisher method (Derkach et al., 2013), and mixed-effects score test (MIST) (Sun et al., 2013) – and Exponential Combination test (Chen et al., 2012) combine burden and variance component tests and hence incorporate both homogeneous and heterogeneous effects of individual genetic variants.

Combined tests are generally more desirable because only mild distributional assumptions are made about the underlying nature of the effects of individual genetic variants on disease, which is largely unknown (Lee et al., 2014). For MIST, two score statistics are derived – one for homogeneous characteristics, and the other for heterogeneous effects – and both are easy to calculate under their respective null hypotheses to test genetic effects on phenotypes. The significance levels of the score statistics are also easy to determine because their respective distributions are known under reasonable parametric assumptions. In addition, since the two score statistics are independent of one another, the resulting p-values of the two score statistics can be combined using simple formulae, which leads to a single significance value. This results in the computational efficiency of MIST. MIST is a very general model that includes burden test and SKAT as special cases. In comparison with MIST, the weighted linear combination of SKAT and burden statistics in Optimal SKAT is less ideal, since the two score statistics are not statistically independent (Sun et al., 2013). For Fisher method and exponential combination test, the estimation of the significance level of the statistics is computationally intensive because the joint distribution of the statistics is not known and permutation analysis is required (Lee et al., 2014).

While genetics has significant contributions to many psychiatric disorders, they are often functional under certain environment. Indeed, G×E has been shown to be relevant for many psychiatric disorders (Cadoret et al., 1983; Milberger et al., 1997; Wahlberg et al., 1997; Yaffe et al., 2000; Eley et al., 2004; Kim-Cohen et al., 2006). Environmental factors, such as disease risk factors, could interact with genetic factors to result in variations in phenotypes. For example, genetics may modulate the effects of various risk factors on the manifestation of a disease, causing varying severities of the disease across individuals even though they may be exposed to the same risk factors. Recently, G×E has been incorporated into burden tests (Jiao et al., 2013) and variance component tests (Lin et al., 2013, 2016; Ge et al., 2015). However, as mentioned earlier, burden tests and variance component tests make assumptions on the underlying nature of the effects of individual genetic variants on disease, and these tests become less powerful when the assumptions do not hold. There is a need to incorporate burden and variance component tests for G×E into one statistical model.

The aim of this study is to extend MIST to examine effects of G×E on neuroimaging phenotypes. We chose MIST because it has been shown to be consistently more powerful than other tests, especially at stringent thresholds, while still controlling for false positive rate (Moutsianas et al., 2015). Another advantage of MIST is its computational efficiency, which is especially important for imaging genetics, since this makes the use of voxelwise neuroimaging measures as phenotypes feasible. Hence, in this study we proposed a set-based MixGE. MixGE is designed to incorporate both fixed and random effects of G×E, to investigate homogeneous and heterogeneous contributions of sets of genetic variants and their interaction with environmental risks to phenotypes. In Section 2, we explained the MixGE model, and how the score statistics for the terms associated with fixed and random effects of G×E were calculated and combined into a single significance value. We then described the simulated data generated to test the type 1 error rate, and power of MixGE as compared to other existing methods. Next we described the real data used in this study. In brief, we used data from the ADNI database, including 21 candidate risk genes for late-onset AD as variant sets, the first principal component of six cardiovascular disease risk factors as an environmental factor, and hippocampal volume and TBM as the neuroimaging phenotypes. In Section 3, we showed the results from testing MixGE on simulated data as well as on the two neuroimaging phenotypes of the ADNI data. Running MixGE on the first neuroimaging phenotype of hippocampal volume serves to replicate the results of the kernel machine method (hereafter referred to as kernel machine method (KMM) for convenience) of (Ge et al., 2015) as a sanity check. Running MixGE on the second neuroimaging phenotype of TBM demonstrated the potential of MixGE for voxelwise neuroimaging phenotypes of imaging genetics. We implemented the MixGE model in MATLAB, and the software and demo are available at http://www.bioeng.nus.edu.sg/cfa/imaginggenetics.html.

## 2. Materials and methods

### 2.1. A set-based mixed effect model for gene-environment interaction (MixGE)

We now introduce a unified set-based mixed effect model for gene-environment interaction (MixGE). We assume that there are *N* unrelated subjects with brain measures, genotyping, environmental factor, and demographic data. Let *y*_*i*_ be a quantitative brain measure for the *i*^*th*^ subject. Let *X*_*i*_ be a *m* × 1 vector of potential confounding covariates for the *i*^*th*^ subject, such as demographic variables and population stratification. For simplicity of notation, we incorporate the intercept into *X*_*i*_. Let *G*_*i*_ be a variant set that is a *p* × 1 vector with the genotypes of *p* SNPs for the *i*^*th*^ subject. Each SNP takes the values of 0 (homozygotic major alleles), 1 (heterozygote), or 2 (homozygotic minor alleles). Let *e*_*i*_ be an environmental risk factor for the *i*^*th*^ subject. Based on the above notation, we define the MixGE model as::

(1) $$\begin{array}{r} g\left\{E\left(y_{i}\right)\right\}=X_{i}^{T}\beta_{x}+e_{i}\beta_{e}+G_{i}^{T}W\pi^{\left(1\right)}+e_{i}G_{i}^{T}W\pi^{\left(2\right)}+e_{i}G_{i}^{T}\delta \;. \end{array}$$

*g*(·) is a link function. When *y*_*i*_ only takes the values of 0 or 1, *g*(·) can be a logit function. When *y*_*i*_ is continuous, *g*(·) can be an identity function. ^*T*^ represents the vector transpose operation. β_*x*_ and β_*e*_ are the regression coefficients for *X*_*i*_ and *e*_*i*_ respectively, where β_*x*_ is a *m* × 1 vector and β_*e*_ is a scalar. *W* is a *p* × *q* weight matrix based on *q* variant characteristics (such as nonsense, missense, insertion, deletion, *etc*) of *p* SNPs, if effects of the various characteristics are expected to be different (Sun et al., 2013). In the simplest case, *W* can be a *p* × 1 vector with elements of 1/*p*, which means that all SNPs are equally weighted according to one common variant characteristic. *W* can also be used to exclude SNPs from the model by giving them a weight of 0. π^(1)^ and π^(2)^ are *q* × 1 vectors and account for fixed effects of the variant set *G*_*i*_, and its interaction with an environmental risk factor *e*_*i*_ respectively. On the other hand, $\delta ={\left[\delta_{1},\delta_{2},\cdots ,\delta_{p}\right]}^{T}$ is a *p* × 1 vector and accounts for random effects of individual SNPs in *G*_*i*_, and their interaction with the environmental risk factor *e*_*i*_, that cannot be explained by the fixed effects. We assume that δ follows a distribution with mean 0 and variance τ^2^.

The MixGE model in Equation (1) is a generalized form of commonly used models such as burden test and SKAT (Morgenthaler and Thilly, 2007; Madsen and Browning, 2009), with the addition of G×E. For instance, if heterogeneous effects of individual SNPs are expected to be subtle, we may assume that τ^2^ = 0. The MixGE model is hence simplified to an interaction model of burden test. In particular, when *W* is set as a *p* × 1 vector of 1/*p*, the MixGE model becomes an interaction model of Cohort Allelic Sum Test (Morgenthaler and Thilly, 2007). Whereas, when *W* is set to be a vector of weights, the MixGE model becomes an interaction model of Weighted Sum Statistic (Madsen and Browning, 2009). On the other hand, if π^(1)^ and π^(2)^ are set to zero, this model is equivalent to an interaction model of SKAT (Wu et al., 2011).

### 2.2. Score test statistics

From the model in Equation (1), our null hypothesis *H*_0_ is no interaction effect between the variant set, *G*_*i*_, and the environmental factor, *e*_*i*_, on brain measure, *y*_*i*_. The examination of this null hypothesis is equivalent to showing that π^(2)^ = 0 and τ^2^ = 0. We use score statistics to test *H*_0_ so that – unlike the likelihood ratio test – there is no need to estimate τ^2^ under the alternative. Estimating τ^2^ can be computationally expensive as it requires *p*-dimensional multiple integration. As the score statistic for π^(2)^ asymptotically follows a normal distribution and the score statistic for τ^2^ asymptotically follows a mixture of chi-square distributions, it is challenging to derive the joint distribution of π^(2)^ and τ^2^, especially when they are correlated. We propose a sequential way to calculate the score statistics for τ^2^ and π^(2)^, which will make them independent of one another and hence make the testing of *H*_0_ feasible. For this, we first derive the score statistic for the variance component under the null hypothesis of τ^2^ = 0 without assuming that π^(2)^ = 0. We then derive the score statistic for π^(2)^ under *H*_0_. This sequential derivation ensures the independence of these two score statistics, which has been proven in Sun et al. (2013).

To simplify notations, we rewrite Equation (1) in the matrix form. By denoting $Y={\left[y_{1},y_{2},\cdots ,y_{N}\right]}^{T}$, $X={\left[X_{1},X_{2},\cdots ,X_{N}\right]}^{T}$, $G={\left[G_{1},G_{2},\cdots ,G_{N}\right]}^{T}$, and $E={\left[e_{1},e_{2},\cdots ,e_{N}\right]}^{T}$, Equation (1) can be rewritten as:

(2) $$\begin{array}{r} g\left\{E\left(Y\right)\right\}=X\beta_{x}+E\beta_{e}+GW\pi^{\left(1\right)}+\text{diag}\left(E\right)GW\pi^{\left(2\right)}+\text{diag}\left(E\right)G\delta \;, \end{array}$$

where diag(·) is the operation of taking the elements in *E* as the diagonal elements of an *N* × *N* matrix. We now show how to compute the score statistics for τ^2^ and π^(2)^.

First, the score statistic for τ^2^ can be calculated as:

(3) $$\begin{array}{r} S_{\tau^{2}}={\left(Y-\hat{\mu }\right)}^{T}\text{ diag}\left(E\right)GG^{T}\text{diag}\left(E\right)\;\left(Y-\hat{\mu }\right)\;, \end{array}$$

where

(4) $$\begin{array}{r} \hat{\mu }=g^{-1}(X{\hat{\beta }}_{x}+E{\hat{\beta }}_{e}+GW{\hat{\pi }}^{\left(1\right)}+\text{diag}\left(E\right)GW{\hat{\pi }}^{\left(2\right)}\;). \end{array}$$

${\hat{\beta }}_{x}$, ${\hat{\beta }}_{e}$, ${\hat{\pi }}^{\left(1\right)}$, and ${\hat{\pi }}^{\left(2\right)}$ are the estimates of β_*x*_, β_*e*_, π^(1)^, and π^(2)^ respectively under the null hypothesis of τ^2^ = 0. These coefficients can be obtained via solving the least-squares linear regression of $g\left\{E\left(Y\right)\right\}=X\beta_{x}+E\beta_{e}+GW\pi^{\left(1\right)}+\text{diag}\left(E\right)GW\pi^{\left(2\right)}$.

Under the null hypothesis of τ^2^ = 0, $S_{\tau^{2}}$ follows a mixture of chi-square distributions $\overset{s}{\underset{i=1}{\sum }}\lambda_{i}\chi_{1,i}^{2}$, where λ_1_≥ … ≥ λ_*s*_ are the non-zero eigenvalues of $P=\hat{D}-\hat{D}M{\left(M^{T}\hat{D}M\right)}^{-1}M^{T}\hat{D}$ (Zhang and Lin, 2003). $\hat{D}=\text{diag}\left({\hat{\sigma_{1}}}^{2},\cdots \;,{\hat{\sigma_{N}}}^{2}\right)$, where ${\hat{\sigma_{i}}}^{2}=\overset{N}{\underset{i=1}{\sum }}{\left(y_{i}-{\hat{\mu }}_{i}\right)}^{2}/N$ when the identity link function is used and ${\hat{\sigma_{i}}}^{2}={\hat{\mu }}_{i}\left(1-{\hat{\mu }}_{i}\right)$ when the logit link function is used. *M* = [*X, E, GW*, diag(*E*)*GW*]. This mixture of chi-square distributions can be approximated using the method in Liu et al. (2009). We denote the p-value of $S_{\tau^{2}}$ as $P_{\tau^{2}}$.

To be more general, when the variances of δ_*j*_ are not expected to be the same for all *j*, we can define the variance as $\omega_{j}\tau^{2}$. The score statistic for $\omega_{j}\tau^{2}$ can be calculated as:

(5) $$\begin{array}{r} \omega S_{\tau^{2}}={\left(Y-\hat{\mu }\right)}^{T}\text{ diag}\left(E\right)G\omega G^{T}\text{diag}\left(E\right)\;\left(Y-\hat{\mu }\right)\;, \end{array}$$

with ω = diag(ω_1_, …, ω_*p*_). For instance, ω_*j*_ can be set to be negatively correlated with MAF (MAF) since by evolutionary theory rarer genetic variants tend to have larger effect sizes.

Second, we compute the score statistic for π^(2)^ as:

(6) $$\begin{array}{r} U_{\pi^{\left(2\right)}}={\left(\text{diag}\left(E\right)GW\right)}^{T}\left(Y-\tilde{\mu }\right)\;, \end{array}$$

where

(7) $$\begin{array}{r} \tilde{\mu }=g^{-1}\left(X{\tilde{\beta }}_{x}+E{\tilde{\beta }}_{e}+GW{\tilde{\pi }}^{\left(1\right)}\right)\;. \end{array}$$

${\tilde{\beta }}_{x}$, ${\tilde{\beta }}_{e}$, and ${\tilde{\pi }}^{\left(1\right)}$ are the estimates of β_*x*_, β_*e*_, and π^(1)^ respectively under the null hypothesis of τ^2^ = 0 and π^(2)^ = 0. These coefficients can be obtained via solving the least-squares linear regression of $g\left\{E\left(Y\right)\right\}=X\beta_{x}+E\beta_{e}+GW\pi^{\left(1\right)}$.

Based on the central limit theory and the law of large number, we can show that $\Sigma^{-1/2}U_{\pi^{\left(2\right)}}$ converges to a *q*-dimensional normal distribution with mean of zero and covariance of identity matrix. $\Sigma ={\left(\text{diag}\left(E\right)GW\right)}^{T}\left\{\tilde{D}-\tilde{D}V{\left(V^{T}\tilde{D}V\right)}^{-1}V^{T}\tilde{D}\right\}\left(\text{diag}\left(E\right)GW\right)$, where *V* = [*X, E, GW*]. $\tilde{D}=\text{diag}\left({\tilde{\sigma_{1}}}^{2},\cdots \;,{\tilde{\sigma_{N}}}^{2}\right)$, where ${\tilde{\sigma_{i}}}^{2}=\overset{N}{\underset{i=1}{\sum }}{\left(y_{i}-{\tilde{\mu }}_{i}\right)}^{2}/N$ when *y*_*i*_ is continuous and ${\tilde{\sigma }}_{i}^{2}={\tilde{\mu }}_{i}\left(1-{\tilde{\mu }}_{i}\right)$ when *y*_*i*_ is binary. To avoid computing the square root of Σ, we compute $U_{\pi^{\left(2\right)}}\Sigma^{-1}U_{\pi^{\left(2\right)}}$ that follows a χ^2^ distribution with *q* degrees of freedom. We denote the p-value of $U_{\pi^{\left(2\right)}}\Sigma^{-1}U_{\pi^{\left(2\right)}}$ as $P_{\pi^{\left(2\right)}}$.

Based on the above construction, the score statistics of $U_{\pi^{\left(2\right)}}$ and $S_{\tau^{2}}$ are statistically independent (Sun et al., 2013). This independence property makes it feasible to combine $U_{\pi^{\left(2\right)}}$ and $S_{\tau^{2}}$ and examine our null hypothesis of τ^2^ = 0 and π^(2)^ = 0. Two possible ways to combine $P_{\pi^{\left(2\right)}}$ and $P_{\tau^{2}}$ are the Fisher and Tippett methods (Koziol and Perlman, 1978). For the Fisher method, *H*_0_ is rejected if $-2\log P_{\pi^{\left(2\right)}}-2\log P_{\tau^{2}}\geq \chi_{4,\alpha }^{2}$. Permutation is not required in this case, unlike in Derkach et al. (2013), because the two score statistics are independent. For the Tippett method, *H*_0_ is rejected if $\min\left(P_{\pi^{\left(2\right)}},P_{\tau^{2}}\right)\leq 1-{\left(1-\alpha \right)}^{1/2}$, where α is the significance level. The Fisher method is more powerful when the alternative hypotheses of both score statistics are likely to be true, whereas the Tippett method is more powerful when the alternative hypothesis of only one of the two score statistics is likely to be true.

### 2.3. Simulation

MixGE was run on simulated data to ensure that it has a reasonable type 1 error rate, and to compare its power against other existing methods. Two separate simulations were carried out for binary and continuous phenotypes. For binary phenotype, simulated data were generated according to the following model adapted from Lin et al. (2013):

(8) $$\begin{array}{r} \;\mathrm{logit}\left(Y_{i}\right)=\log\frac{0.01}{0.99}+0.64E_{i}+\overset{8}{\underset{j=1}{\sum }}a_{j}G_{ij}+\overset{8}{\underset{j=1}{\sum }}b_{j}E_{i}G_{ij} \end{array}$$

where *i* refers to the *i*^*th*^ subject, *j* refers to the *j*^*th*^ variant of the genetic set, *E*_*i*_ ~ N(0, 1) is an environmental factor, and *G*_*ij*_ is a genetic set generated under Hardy-Weinberg equilibrium with MAF ~ U(0, 0.5). How *a*_*j*_ and *b*_*j*_ were set differs depending on whether the simulation is for type 1 error or for power, and will be explained accordingly in the respective subsections 2.3.1 and 2.3.2. The constant coefficients are set to be the same as Lin et al. (2013). *Y*_*i*_ can be generated by taking the logistic of the right hand side of Equation (8), and using that as the probability of success for a Bernoulli trial. Success would represent a disease case subject, whereas failure would represent a control subject. Simulated subjects were generated until there were sufficient cases and controls.

For continuous phenotype, simulated data were generated according to the following model modified from Equation (8):

(9) $$\begin{array}{r} \;Y_{i}=0.64E_{i}+\overset{8}{\underset{j=1}{\sum }}a_{j}G_{ij}+\overset{8}{\underset{j=1}{\sum }}b_{j}E_{i}G_{ij}+\epsilon \end{array}$$

where ϵ is noise with distribution N(0, σ^2^). The intercept term log(0.01/0.99) found in Equation (8) was removed for Equation (9) as it models disease prevalence and is thus meaningless for the continuous phenotype, although the simulation results would not have been affected even if it were included.

Simulated data were generated separately for small samples with 200 subjects (100 disease cases and 100 controls) as well as large samples with 2000 subjects (1000 disease cases and 1000 controls). The reason for simulating small samples is to examine the behavior of MixGE when the number of subjects available is small, which is typical for imaging genetics studies. For every simulated scenario, 10,000 simulations were run.

#### 2.3.1. Type 1 error simulation

For type 1 error simulation, *a* was fixed as [1, 1, −1, −1, 0, 0, 0, 0]^*T*^ while *b* was fixed as zero meaning that there is no G×E effect. *G*_*ij*_ and *E*_*i*_ were regenerated for each simulation. To determine type 1 error rate, p-values for the G×E effect for 10,000 simulations were calculated. The p-values were then thresholded at a significance level of 0.05. Type 1 error rate is the proportion of simulations that pass the significance threshold, even though there is no G×E effect, out of the total 10,000 simulations run.

#### 2.3.2. Power simulation

For power simulation, MixGE was compared against two existing methods: set-based gene-environment interaction test (SBERIA) (Jiao et al., 2013) and KMM (Ge et al., 2015). These two methods were chosen as they perform set-based G×E burden and variance component tests respectively. These methods are hence good representatives of their respective class of methods, and are good bases of comparison for MixGE which is a combination test. *a*_*j*_ ~ Bernoulli(0.5) × [1−2 × Bernoulli(0.5)]. Three different scenarios were tested in the power simulation, each with a different *b*. *b* was fixed as *C* × [1, 1, 1, 1, 1, 1, 1, 1]^*T*^ for the burden scenario, as *C* × [1, 1, −1, −1, 0, 0, 0, 0]^*T*^ which has zero mean for the variance component scenario, and as *C* × [1, 1, 0, 0, 0.5, 0.5, 0.5, 0.5]^*T*^ for the mixture of burden and variance component scenario. For binary phenotype, *C* is given an arbitrary range of positive values such that the simulation is within reasonable power range. For continuous phenotype, *C* is fixed as 1 while varying the variance of the Gaussian noise. *a*_*j*_, *G*_*ij*_, and *E*_*i*_ were regenerated for each simulation. Power in this simulation is defined as the rate at which the various methods are able to significantly detect G×E effect, *i.e*. the proportion of simulations that pass the significance threshold of 0.05 out of the total 10,0000 simulations run for each *C* for each scenario.

### 2.4. ADNI data

The neuroimaging data – including hippocampal volumes and TBM – genetic data, environmental factors, demographic data are the same as those used in previous studies (Stein et al., 2010; Hibar et al., 2011; Ge et al., 2012, 2015). We describe the genetic and neuroimaging data, and their preprocessing steps below.

The data used in this study were obtained from the ADNI database http://adni.loni.usc.edu/. ADNI was launched in 2003 by the National Institute of Aging (NIA), the National Institute of Biomedical Imaging and Bioengineering (NIBIB), the Food and Drug Administration (FDA), private pharmaceutical companies and non-profit organizations, as a $60 million, 5-year public private partnership. The primary goal of ADNI has been to test whether biological markers such as serial magnetic resonance imaging (MRI) and positron emission topography (PET), and clinical and neuropsychological assessments can be combined to measure the progression of mild cognitive impairment (MCI) and early AD. Determination of sensitive and specific markers of very early AD progression is intended to aid researchers and clinicians to develop new treatments and monitor their effectiveness, as well as lessen the time and cost of clinical trials. ADNI is the result of efforts of many coinvestigators from a broad range of academic institutions and private corporations, and subjects have been recruited from over 50 sites across the U.S. and Canada. The initial goal of ADNI was to recruit 800 adults, ages 55 to 90, to participate in the research approximately 200 cognitively normal older individuals to be followed for 3 years, 400 people with MCI to be followed for 3 years, and 200 people with early AD to be followed for 2 years (see http://www.adni-info.org/ for up-to-date information). The data were analyzed anonymously, using publicly available secondary data from the ADNI study, therefore no ethics statement is required for this work.

This study only included 697 subjects (age: 55–91 years) with MRI scans and genotype, environmental and demographic data. Among them, there were 295 females and 402 males. The amount of education received by these subjects ranged from 4 to 20 years.

#### 2.4.1. Environmental factors

The environmental factors used in this and previous studies (Ge et al., 2015) are six cardiovascular disease risk variables, including age, gender, body mass index, systolic blood pressure, current smoking status and diabetes. These cardiovascular disease risk factors were chosen as environmental factors because sufficient evidence suggests that they increase the risk for AD and accelerate AD progression (Kivipelto et al., 2001; Luchsinger et al., 2005). For gender, females were given a value of 1 whereas males were given a value of 0. For current smoking status, smokers were given a value of 1 whereas non-smokers were given a value of 0. For diabetes, diabetics were given a value of 1 whereas non-diabetics were given a value of 0. Here, we performed principal component analysis on these six environmental variables. The first principal component accounted for 81.9% of the total variation of the six environmental variables. It was used as the environmental factor for MixGE, as MixGE is only able to accommodate one environmental factor. The loadings of the six cardiovascular disease risk variables on the first principal component are 0.213, –0.522, 0.548, 0.301, 0.537, and 0.0424 respectively.

#### 2.4.2. Genotyping and variant sets

The genome-wide SNP data was preprocessed according to the ENIGMA2 1KGP cookbook (v3): http://enigma.ini.usc.edu/wp-content/uploads/2012/07/ENIGMA2_1KGP_cookbook_v3.pdf. Twenty-one candidate genes that were previously identified as risk genes for AD (Lambert et al., 2013; Lu et al., 2014) were used in this study. These sets of genetic variants were also previously used to examine the G×E influence on the aging brain (Ge et al., 2015). The SNPs on the coding regions, as well as 20kb upstream and downstream of these 21 genes were extracted. The names of the 21 genes and the number of SNPs for each are listed in Table 1. The SNPs for each gene were considered as a separate set of variants. Each of the 21 candidate risk genes were put through MixGE separately.

**Table 1:** **Twenty one candidate risk genes for Alzheimer's disease (AD)**.

**Chromosome**	**Gene**	**Number of SNPs**
19	*ABCA7*	240
2	*BIN1*	301
20	*CASS4*	165
6	*CD2AP*	421
19	*CD33*	85
11	*CELF1*	97
8	*CLU*	116
1	*CR1*	264
18	*DSG2*	219
7	*EPHA1*	115
14	*FERMT2*	242
6	*HLA-DRB5*	62
2	*INPP5D*	495
5	*MEF2C*	272
11	*MS4A6A*	63
11	*PICALM*	360
8	*PTK2B*	419
4	*REST*	146
14	*SLC24A4*	716
11	*SORL1*	233
7	*ZCWPW1*	74

#### 2.4.3. MRI acquisition and analysis

All ADNI 1.5T structural brain MRI scans were processed using FreeSurfer (Dale et al., 1999). Intra-cranial volume and bilateral hippocampal volumes were automatically computed using FreeSurfer after skull stripping, B1 bias field correction, segmentation, and labeling (Fischl et al., 2002), and passed rigorous visual quality control checks (Ge et al., 2015).

The process used to generate the TBM data was described in (Hua et al., 2008), and is restated here. First, a random subset of healthy elderly subjects were chosen. The intensities of their MRI scans were normalized, aligned using a 9 parameter affine transform, and averaged voxelwise to create an initial affine average template. The scans of this subset of subjects were then warped to the affine average template using a non-linear inverse consistent elastic intensity-based registration algorithm (Leow et al., 2005), and averaged to create a non-linear average intensity template. Inverse geometric centering of the displacement fields to the non-linear average intensity template was performed to construct the minimal deformation template (MDT). The MDT serves as an unbiased atlas image to which all other images were transformed using the same non-linear inverse consistent elastic intensity-based registration algorithm as earlier (Leow et al., 2005). The determinant of the Jacobian matrix of the deformation was computed to assess volumetric tissue difference at each voxel, which encodes local volume excess or deficit relative to the atlas image. This volumetric tissue difference relative to the atlas at each voxel was used as a quantitative measure of brain tissue volume difference for examining the MixGE. From here on, we shall refer to “the determinant of the Jacobian matrix of the deformation” as “local volume change.” There are a total of 2,061,878 voxels per image.

### 2.5. MixGE application to hippocampal volume and TBM

We applied MixGE to examine the interactive effect of cardiovascular risks and AD candidate genes on hippocampal volume and TBM of the brain using the aforementioned ADNI dataset. For this, the environmental factor was defined as the first principal component of the six cardiovascular factors mentioned earlier. The genes are listed in Table 1.

For TBM, we applied MixGE to the local volume change measure and examined the null hypothesis *H*_0_, at the voxel-level of the atlas. We then performed false discovery rate (FDR) adjustment on the obtained *p*-values, according to the procedure outlined in Storey (2002). Lastly we thresholded the adjusted *p*-values at a significance level of 0.05.

## 3. Results and discussion

In this section, we first present the results of the simulated data to evaluate the type 1 error rate of MixGE and to compare its power with two existing techniques: SBERIA (Jiao et al., 2013) and KMM (Ge et al., 2015).

For real ADNI data, we run MixGE on hippocampal volume to compare with the results obtained by Ge et al. (2015) using KMM. We then run MixGE on TBM to demonstrate the potential of MixGE for voxelwise imaging genetics. In both experiments, intra-cranial volume and education were considered as covariates in the MixGE model, where intra-cranial volume was automatically calculated by FreeSurfer. Hence, in the MixGE model, *X* is a (697 × 3) matrix with the first column of constant, the second column of intra-cranial volume, and the third column of education. *W* is a *p* × 1 vector with elements of 1/*p*.

### 3.1. Simulation results

#### 3.1.1. Type 1 error simulation

According to the simulated data for binary phenotype, MixGE had type 1 error rates of 0.0479 and 0.0507 for small samples of 200 subjects and large samples of 2000 subjects respectively. The type 1 error rates for SBERIA (Jiao et al., 2013) were determined to be 0.0523 and 0.0504 for small samples of 200 subjects and large samples of 2000 subjects respectively, while that of KMM (Ge et al., 2015) were determined to be 0.0287 and 0.0751 respectively.

**Figure 1:**
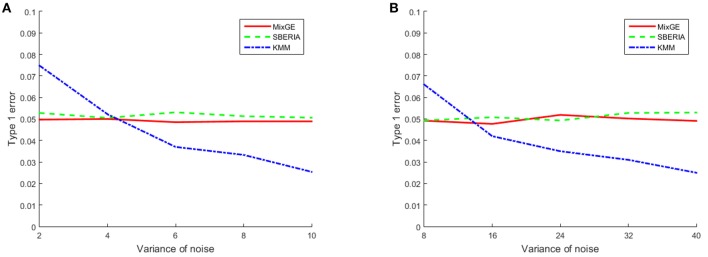
**Results of the type 1 error simulation for continuous phenotype**. Type 1 error of mixed effect model for gene-environment interaction (MixGE) (red solid) as compared to set-based gene-environment interaction test (SBERIA) (Jiao et al., 2013) (green dashed) and kernel machine method (KMM) (Ge et al., 2015) (blue dot-dashed) at various variance of Gaussian noise added. (A, B) Are for small samples of 200 subjects and large samples of 2,000 subjects, respectively.

The results for the type 1 error simulation for continuous phenotype is shown in Figure 1. The type 1 error rates for both MixGE and SBERIA (Jiao et al., 2013) are close to 0.05 regardless of the variance of Gaussian noise added. The type 1 error rates for KMM (Ge et al., 2015) is higher than 0.05 when variance of Gaussian noise added is low, and drops below 0.05 when variance of Gaussian noise added is high. Sample size appear to have little effect on type 1 error rates for continuous phenotype.

These results show that MixGE is a valid method that does not have an inflated false positive rate.

#### 3.1.2. Power simulation

**Figure 2:**
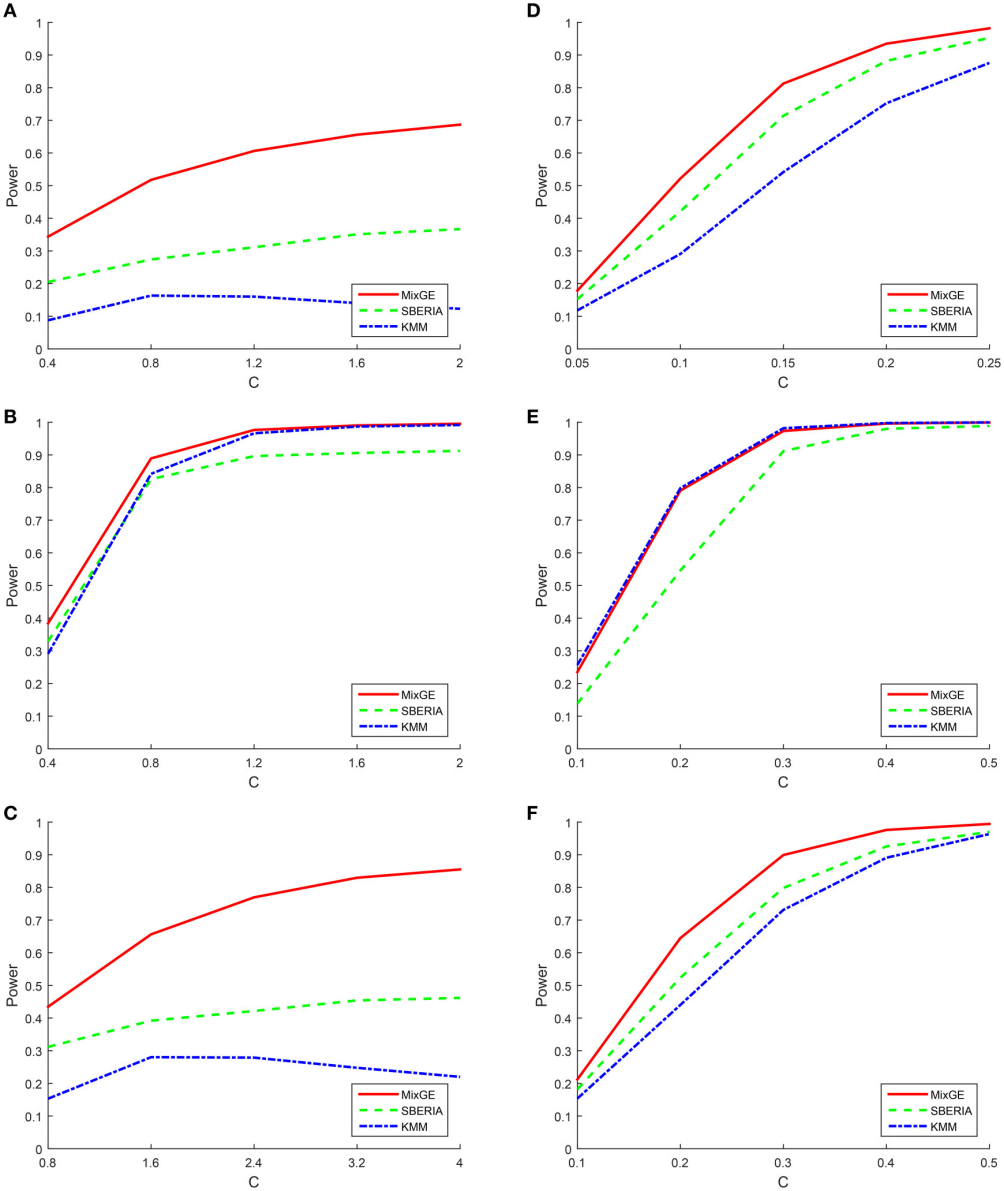
**Results of the power simulation for binary phenotype**. Power of mixed effect model for gene-environment interaction (MixGE) (red solid) as compared to set-based gene-environment interaction test (SBERIA) (Jiao et al., 2013) (green dashed) and kernel machine method (KMM) (Ge et al., 2015) (blue dot-dashed) at various values of *C*. **(A, D)** Are for the burden scenario for small samples of 200 subjects and large samples of 2,000 subjects, respectively. **(B, E)** Are for the variance component scenario for small samples and large samples, respectively. **(C, F)** Are for the mixture of burden and variance component scenario for small samples and large samples, respectively.

For binary phenotype, *C* is given an arbitrary range of positive values such that the simulation is within a reasonable power range. For the burden scenario, *C* ∈ {0.4, 0.8, 1.2, 1.6, 2} for small samples of 200 subjects and *C* ∈ {0.05, 0.1, 0.15, 0.2, 0.25} for large samples of 2000 subjects. For the variance component scenario, *C* ∈ {0.4, 0.8, 1.2, 1.6, 2} for small samples and *C* ∈ {0.1, 0.2, 0.3, 0.4, 0.5} for large samples. For the mixture of burden and variance component scenario, *C* ∈ {0.8, 1.6, 2.4, 3.2, 4} for small samples and *C* ∈ {0.1, 0.2, 0.3, 0.4, 0.5} for large samples. The power of MixGE as compared to SBERIA (Jiao et al., 2013) and KMM (Ge et al., 2015) for these three scenarios against these various values of *C* are plotted in Figure 2.

Figure 2 shows that in general, when the number of subjects is small, *C* has to be much larger in order to detect the G×E effect. Also, when the number of subjects is small, all three methods suffer great statistical power loss for the burden scenario. Furthermore, the power for KMM (Ge et al., 2015) begins to decrease when *C* becomes larger than 0.8. This is probably because of increase in the estimated variance of *b* as *C* increases, thereby causing power loss. The same trends but to a smaller extent is also observed for the mixture of burden and variance component scenario, as expected.

We indeed see that SBERIA (Jiao et al., 2013) and KMM (Ge et al., 2015) performed best for the scenarios that they were designed for (burden and variance component respectively) but performed worst for the opposite scenario (variance component and burden respectively). They both performed somewhat average for the mixture of burden and variance component scenario.

MixGE on the other hand performs consistently well for all three scenarios. For the simulated data with small samples, MixGE outperformed the other two methods for all scenarios. It is especially notable, that while MixGE also suffered significant power loss for the burden scenario, it is by far the least affected by small sample size as compared to the other two methods. For the simulated data with large samples, MixGE has more power than the other two methods for the burden and the mixture of burden and variance component scenarios. For the variance component scenario, the performance of MixGE and KMM (Ge et al., 2015) are very close. KMM (Ge et al., 2015) slightly outperforms MixGE when the effect size is smaller, whereas MixGE outperforms KMM (Ge et al., 2015) when the effect size is larger. This power simulation shows the strength of MixGE. MixGE exhibits relatively high power whether for datasets with small or large sample sizes. MixGE also performs well for all three scenarios, which shows that MixGE can be applied to any dataset without having to first make any assumption on the underlying nature of the G×E effects.

**Figure 3:**
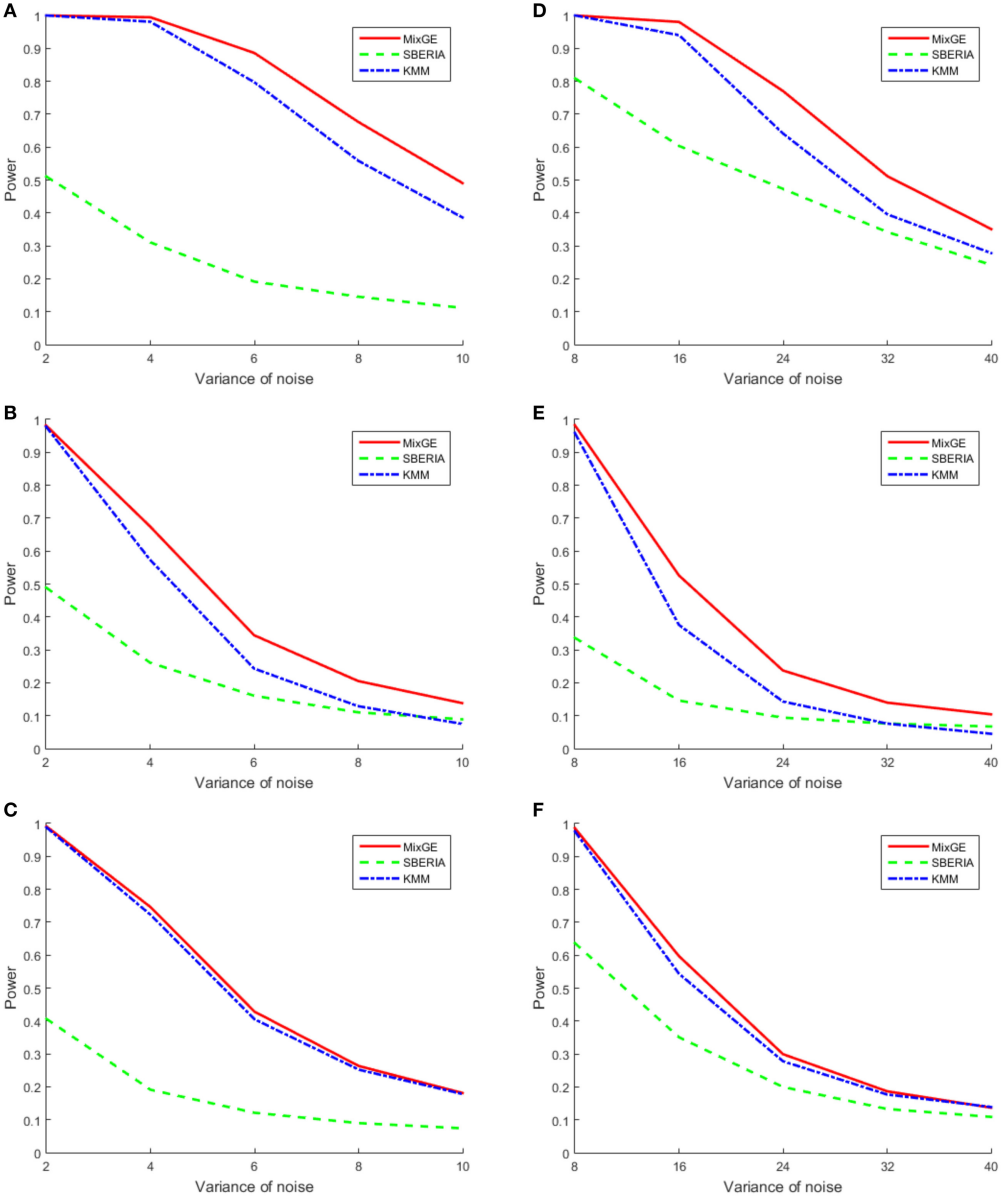
**Results of the power simulation for continuous phenotype**. Power of mixed effect model for gene-environment interaction (MixGE) (red solid) as compared to set-based gene-environment interaction test (SBERIA) (Jiao et al., 2013) (green dashed) and kernel machine method (KMM) (Ge et al., 2015) (blue dot-dashed) at various variance of Gaussian noise added. **(A, D)** Are for the burden scenario for small samples of 200 subjects and large samples of 2,000 subjects, respectively. **(B, E)** Are for the variance component scenario for small samples and large samples, respectively. **(C, F)** Are for the mixture of burden and variance component scenario for small samples and large samples, respectively.

For continuous phenotype, variance of Gaussian noise added σ^2^ ∈ {2, 4, 6, 8, 10} for small samples of 200 subjects and σ^2^ ∈ {8, 16, 24, 32, 40} for large samples of 2000 subjects. The power of MixGE as compared to SBERIA (Jiao et al., 2013) and KMM (Ge et al., 2015) for these three scenarios against these various values of σ^2^ are plotted in Figure 3.

Figure 3 shows that in general, when the number of subjects is small, σ^2^ has to be smaller in order to detect the G×E effect. In all scenarios, MixGE and KMM (Ge et al., 2015) performed similarly, with MixGE having slightly more power than KMM (Ge et al., 2015). On the other hand, SBERIA (Jiao et al., 2013) performed poorly as compared to the other two methods.

### 3.2. Hippocampal volume

In this experiment, we employed MixGE and KMM (Ge et al., 2015) to investigate the interactive effect of individual variant sets listed in Table 1 with cardiovascular risks on hippocampal volume. *Y* therefore represents hippocampal volume. For MixGE the first principal component of the six cardiovascular risk factors was used as the environmental factor, as MixGE is only able to accommodate one environmental factor. Columns of *P*_*Fisher*_ and *P*_*Tippett*_ in Table 2, respectively, show the results from the MixGE model based on the Fisher and Tippett methods for combining the p-values of the two score statistics. Additionally, in Table 2, column $P_{\pi^{\left(2\right)}}$ is equivalent to a burden test with G×E, and column *P*_*Random*_ is equivalent to SKAT with G×E. On the other hand, the column *P*_*all*_ shows the results of KMM (Ge et al., 2015) with all six cardiovascular risk factors as environmental factors, and column *P*_*PC*_ shows the results of KMM (Ge et al., 2015) with the first principal component as the only environmental factor.

**Table 2:** **Results of testing mixed effect model for gene-environment interaction (MixGE) with hippocampal volume as phenotype**.

**Gene**	**Proposed method**					**KMM (Ge et al., 2015)**	
	**$P_{\pi^{\left(2\right)}}$**	**$P_{\tau^{2}}$**	***P*****_*Fisher*_**	***P*****_*Tippett*_**	***P*****_*random*_**	***P*****_*all*_**	***P*****_*PC*_**
*ABCA7*	1.87E-01	4.65E-01	2.99E-01	3.39E-01	1.82E-01	4.79E-01	1.00E00
*BIN1*	2.88E-01	5.14E-01	4.31E-01	4.93E-01	4.84E-01	1.67E-01	1.00E00
*CASS4*	5.56E-01	6.60E-01	7.35E-01	8.03E-01	8.28E-01	4.92E-01	1.00E00
*CD2AP*	6.26E-01	3.76E-01	5.75E-01	6.10E-01	6.60E-01	9.54E-01	1.00E00
*CD33*	5.40E-01	1.64E-01	3.03E-01	3.00E-01	3.07E-01	8.45E-01	1.00E00
*CELF1*	8.52E-01	3.92E-02	1.47E-01	7.69E-02	7.08E-02	2.84E-01	1.00E00
*CLU*	4.78E-01	6.53E-01	6.76E-01	7.28E-01	6.87E-01	6.02E-01	1.00E00
*CR1*	2.05E-02	1.31E-02	2.48E-03	2.60E-02	**1.35E-03**	**4.85E-04**	4.90E-01
*DSG2*	4.47E-01	1.59E-01	2.59E-01	2.93E-01	2.47E-01	5.66E-01	1.00E00
*EPHA1*	2.78E-01	**3.59E-05**	**1.25E-04**	**7.17E-05**	**4.32E-04**	**5.64E-04**	6.70E-01
*FERMT2*	7.79E-01	9.71E-01	9.68E-01	9.51E-01	9.98E-01	3.42E-01	1.00E00
*HLA-DRB5*	3.85E-01	9.37E-01	7.28E-01	6.22E-01	3.58E-01	7.59E-01	1.00E00
*INPP5D*	2.19E-02	5.66E-01	6.67E-02	4.32E-02	2.00E-01	5.27E-01	1.00E00
*MEF2C*	4.43E-01	4.43E-01	5.15E-01	6.89E-01	3.62E-01	5.80E-02	1.00E00
*MS4A6A*	9.46E-01	6.20E-01	8.99E-01	8.55E-01	1.00E00	9.02E-01	1.00E00
*PICALM*	3.91E-01	2.05E-01	2.82E-01	3.68E-01	2.94E-01	6.79E-01	1.00E00
*PTK2B*	3.93E-01	2.73E-01	3.46E-01	4.71E-01	4.56E-01	4.60E-01	1.00E00
*REST*	5.64E-01	6.13E-01	7.13E-01	8.10E-01	5.16E-01	1.89E-01	1.00E00
*SLC24A4*	2.15E-01	2.13E-01	1.87E-01	3.81E-01	8.84E-02	2.11E-01	1.00E00
*SORL1*	4.90E-01	6.39E-01	6.77E-01	7.40E-01	8.01E-01	9.19E-01	1.00E00
*ZCWPW1*	4.14E-01	7.82E-01	6.89E-01	6.57E-01	4.53E-01	2.65E-01	1.00E00

*The numerical values are p-values, and the significant p-values after Bonferroni correction (<0.00238) are in bold. The fifth column P_random_ under the heading of “Proposed method” is the p-values of the score statistics of random effects without taking into account fixed effects, which is equivalent to SKAT (SKAT) with G×E (G×E). It is a special case of MixGE and is shown here for illustration purposes only, and is not used in the computation of P_Fisher_ or P_Tippett_. The first column P_all_ under the heading of “KMM (KMM)” is the p-values obtained using Ge et al. (2015) with all six cardiovascular disease risk factors as environmental factors, while the second column is obtained using only the first principal component of the six cardiovascular risk factors*.

From Table 2, SKAT with G×E, KMM (Ge et al., 2015), and MixGE, revealed the significant interactive effect of *EPHA1* and the cardiovascular risks on the hippocampal volume with the smallest p-value given by MixGE. SKAT and KMM (Ge et al., 2015) also revealed the significant interactive effect of *CR1* with the cardiovascular risks on hippocampal volume. However, MixGE failed to detect such an interactive effect after Bonferroni correction. As mentioned earlier, the MixGE method is most powerful when the G×E comprises a good mixture of fixed and random effects. If the interaction is predominantly of random effects, then MixGE could become weaker than the variance component methods. This is a slight limitation of MixGE, in exchange for the benefit of not having to make any assumptions of the effects of G×E on phenotypes, which is largely unknown. Another point to note is that MixGE is only able to accommodate one environmental factor, which in this case is the first principal component of the six cardiovascular risk factors that only accounts for 81.9% of the total variation of all six factors. Weaker statistical power in the MixGE method could be due to loss of variance in the environmental factor, which is a limitation of the MixGE method in comparison with KMM (Ge et al., 2015). When the first principal component was used as the only environmental factor for KMM (Ge et al., 2015), no significant results were obtained at all.

### 3.3. TBM

In this experiment, we employed MixGE to examine the interactive effect of individual variant sets listed in Table 1 with cardiovascular risks on TBM. In particular, we describe our findings using the Fisher combined score method below. It took < 7 min to perform the computation for each variant set on a consumer grade laptop. On the other hand, it would take an estimated 90 days to perform the same computation using KMM (Ge et al., 2015).

**Figure 4:**
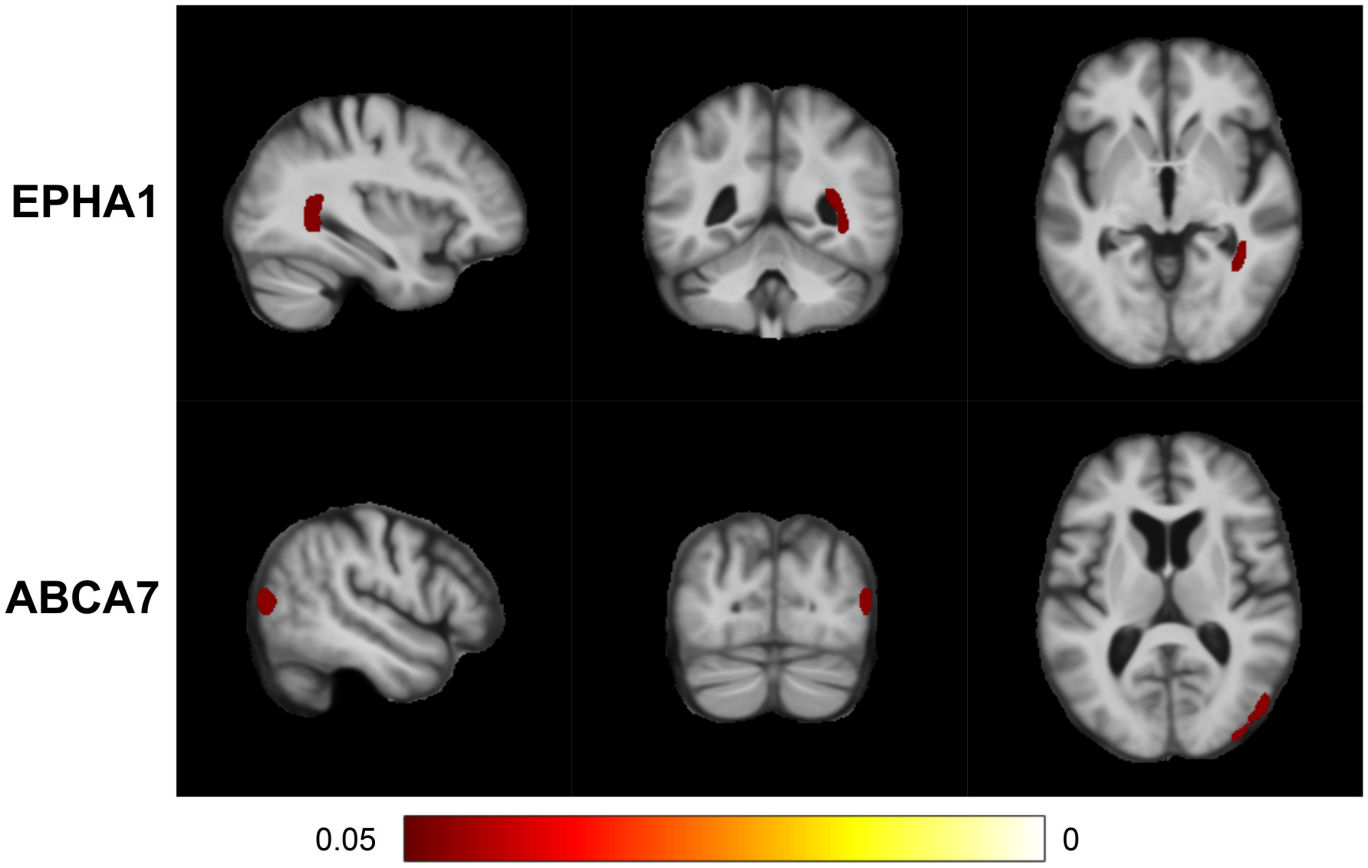
**False discovery rate (FDR) adjusted statistical maps for the effect of gene-environment interaction (G×E) on tensor-based morphometry (TBM)**. From top to bottom, the panels, respectively, show the statistical maps for the variant sets of *EPHA1* and *ABCA7*.

MixGE first discovered the interactive effect of *EPHA1* and cardiovascular risk on local volume change of the right inferior lateral ventricle and the right posterior hippocampus (Table 3). The TBM analysis provided additional information of anatomical location and demonstrated the coherent influence of the G×E on these two adjacent structures (Figure 4). This finding is in line with the result on hippocampal volume, suggesting the robustness and statistical power of the MixGE model. This finding is also consistent with previous finding of the effect of *EPHA1* on hippocampal volume in MCI (Wang et al., 2015).

**Table 3:** **Significant clusters of gene-environment interaction (G'E) on tensor-based morphometry (TBM), the brain regions in which the clusters were found, and the sizes of the clusters**.

**Gene**	**Brain region**	**Cluster size**
*EPHA1*	Right inferior lateral ventricle and posterior hippocampus	2,089
*ABCA7*	Right superior parietal cortex	2,387

*Significant clusters were found for the variant sets of EPHA1 and ABCA7 after FDR (FDR) correction*.

MixGE further revealed the interactive effect of *ABCA7* and cardiovascular risk on local volume change of the right superior parietal cortex (Table 3). *ABCA7* (ATP-binding cassette sub-family A member 7) is a transmembrane transporter that mediates the biogenesis of high-density lipoprotein (Tanaka et al., 2011), and has the capacity to stimulate cellular cholesterol efflux and regulate amyloid precursor protein processing resulting in an inhibition of β-amyloid production (Chan et al., 2008). *ABCA7* is involved in lipid metabolism and transport, and is a possible link between cardiovascular risk factors and AD (Stampfer, 2006; Jones et al., 2010; Reitz et al., 2013). Furthermore, *ABCA7* has been shown to be nominally associated with the brain atrophy in the posterior portion of the cerebral cortex (Carrasquillo et al., 2014), which is consistent with the significant cluster identified in this study.

## 4. Conclusions

In summary, we proposed the MixGE method to examine the effect of G×E on neuroimaging phenotypes. In particular, the MixGE model incorporates both fixed and random effects of the G×E, which is superior to burden and variance component tests, since for MixGE no assumptions have to be made of the effects of G×E on phenotypes, which is largely unknown. We evaluated MixGE with simulated data and showed that it controlled well for type 1 error. MixGE also had more statistical power than the other two methods that were compared, SBERIA (Jiao et al., 2013) and KMM (Ge et al., 2015), in all simulated scenarios. Furthermore, KMM (Ge et al., 2015) did not seem to control well for type 1 error. We also demonstrated the use of MixGE on real ADNI data with brain structural volumes and TBM as phenotypes. Our results showed consistent findings across the hippocampal volume and TBM measures, suggesting the robustness of the MixGE model. MixGE further revealed the interactive effect of *ABCA7* and cardiovascular risk on local volume change of the right superior parietal cortex. This is a potential new discovery that warrants further investigation. The computational efficiency of MixGE made it feasible for MixGE to be used on voxelwise phenotypes such as TBM in the first place. It took < 7 min to perform the computation for each variant set on a consumer grade laptop. On the other hand, it would take an estimated 90 days to perform the same computation using KMM (Ge et al., 2015).

The MixGE model proposed in this study can only accommodate one environmental factor for a gene set and environment interaction, which is less flexible in comparison with KMM (Ge et al., 2015). Nevertheless, for clinical applications, it is often important to understand how individual environmental factors interact with gene on clinical phenotypes rather than all together.

## Author contributions

CW, AQ, JS, and CG contributed to the method derivation and implementation. CW, BG, and AQ also contributed to the experiment design and implementation. TG and DH contributed to the experiment neuroimage analysis. All authors contributed to the manuscript writing.

## Funding

This work was supported by the Singapore National Medical Research Council [NMRC/TCR/004-NUS/2008; NMRC/TCR/012-NUHS/2014; NMRC/CBRG/0039/2013], and the Canadian Institutes of Health Research [MOP-115110]. Data collection and sharing for this project was funded by the Alzheimer's Disease Neuroimaging Initiative (ADNI) (National Institutes of Health Grant U01 AG024904) and DOD ADNI (Department of Defense award number W81XWH-12-2-0012). ADNI is funded by the National Institute on Aging, the National Institute of Biomedical Imaging and Bioengineering, and through generous contributions from the following: AbbVie, Alzheimers Association; Alzheimers Drug Discovery Foundation; Araclon Biotech; BioClinica, Inc.; Biogen; Bristol-Myers Squibb Company; CereSpir, Inc.; Cogstate; Eisai Inc.; Elan Pharmaceuticals, Inc.; Eli Lilly and Company; EuroImmun; F. Hoffmann-La Roche Ltd and its affiliated company Genentech, Inc.; Fujirebio; GE Healthcare; IXICO Ltd.; Janssen Alzheimer Immunotherapy Research & Development, LLC.; Johnson & Johnson Pharmaceutical Research & Development LLC.; Lumosity; Lundbeck; Merck & Co., Inc.; Meso Scale Diagnostics, LLC.; NeuroRx Research; Neurotrack Technologies; Novartis Pharmaceuticals Corporation; Pfizer Inc.; Piramal Imaging; Servier; Takeda Pharmaceutical Company; and Transition Therapeutics. The Canadian Institutes of Health Research is providing funds to support ADNI clinical sites in Canada. Private sector contributions are facilitated by the Foundation for the National Institutes of Health (www.fnih.org). The grantee organization is the Northern California Institute for Research and Education, and the study is coordinated by the Alzheimer's Therapeutic Research Institute at the University of Southern California. ADNI data are disseminated by the Laboratory for Neuro Imaging at the University of Southern California.

### Conflict of interest statement

The authors declare that the research was conducted in the absence of any commercial or financial relationships that could be construed as a potential conflict of interest.

## Abbreviations

AD: Alzheimer's disease
ADNI: Alzheimer's Disease Neuroimaging Initiative
FDA: Food and Drug Administration
FDR: False discovery rate
GWAS: genome-wide association studies
G × E: gene-environment interaction
KMM: kernel machine method
MAF: minor allele frequency
MCI: mild cognitive impairment
MDT: minimal deformation template
MiST: mixed-effects score test
MixGE: mixed effect model for gene-environment interaction
MRI: magnetic resonance imaging
NIA: National Institute of Aging
NIBIB: National Institute of Biomedical Imaging and Bioengineering
PET: positron emission topography
SBERIA: set-based gene-environment interaction test
SKAT: sequence kernel association test
SNP: single nucleotide polymorphism
TBM: tensor-based morphometry.
